# The effects of remifentanil-propofol combined with dexmedetomidine on cognitive dysfunction in elderly patients after ureteroscopic holmium laser lithotripsy: a double-blind randomized controlled trial

**DOI:** 10.1186/s13063-022-06121-2

**Published:** 2022-03-03

**Authors:** Fangjun Wang, Dan Xie, Hongchun Xu, Qin Ye, Le Wu, Xiao Pei Gao

**Affiliations:** Department of Anesthesiology, Affiliated Hospital, North Sichuan Medical College, Nanchong, 637000 China

**Keywords:** Dexmedetomidine, Remifentanil, Propofol, Cognitive dysfunction, Elderly

## Abstract

**Background:**

A clinical study indicated that infusion of dexmedetomidine without a loading dose administered intraoperatively provided a smooth and hemodynamically stable emergence and improved the quality of recovery with fewer postoperative side effects and reduced analgesic requirements. The objective was to determine whether administering remifentanil-propofol combined with dexmedetomidine during general anesthesia would decrease the incidence and severity of postoperative emergence agitation, anxiety, and depression without affecting cognitive dysfunction in elderly patients.

**Methods:**

A total of 120 elderly patients scheduled for ureteroscopic holmium laser lithotripsy were randomly allocated to the PR group and administered normal saline, and the PRD group was administered dexmedetomidine 0.4 μg kg^−1^ h^−1^ intravenously after the induction of anesthesia and stopped 30 min before the end of surgery. The primary outcome was the Mini-Mental State Examination score. The secondary outcomes were the Richmond Agitation Sedation, the State-Trait Anxiety Inventory, and the Zung Self-Rating Depression Scale scores; the memory span for Arabic numerals; the duration of surgery; and the time to spontaneous respiration, recovery, and extubation.

**Results:**

The MMSE scores were lower at T_1–2_ in the two groups (*P <* 0.001). The dosage of propofol and remifentanil decreased more significantly in the PRD group than in the PR group (*P <* 0.001). Both the RASS scores and the incidence of emergence agitation (EA) in the PRD group were significantly lower than those in the PR group at t_1–3_ (*P <* 0.001). Compared to the PR group, the ZSDS scores and STAI scores at T_1–2_ were lower in the PRD group (*P* < 0.005). The number of the Arabic numbers that were accurately recalled from memory was lower at T_2_ in the PR group than in the PRD group (*P <* 0.001).

**Conclusion:**

Dexmedetomidine administration has no influence on postoperative cognitive dysfunction but could reduce both the dosage of remifentanil and propofol needed during ureteroscopic holmium laser lithotripsy and the incidence and severity of postoperative emergence agitation, anxiety, and depression in elderly patients.

**Trial registration:**

Chinese Clinical Trial Registry ChiCTR1900021254. Registered on 3 February 2019

## Introduction

Postoperative cognitive dysfunction (POCD) is a common postoperative complication that adversely affects the patients’ social independence, quality of life, and mortality [1]. Approximately 12% of patients with healthy cognitive function undergoing anesthesia and noncardiac surgery will develop symptoms of cognitive dysfunction after their procedure [2]. In particular, the incidence of POCD is much higher for elderly surgical patients [3]. Risk factors, including preoperative impairment in neurocognitive function, advanced age, metabolic disturbances, duration/type of surgery, hypoxemia, use of certain anesthetics, and pain, are implicated in contributing to POCD [4]. As there is no effective treatment for POCD, the prevention or reduction of POCD incidence is more important.

The incidence of POCD was significantly higher in elderly patients undergoing laparoscopic cholecystectomy anesthetized with sevoflurane or isoflurane compared to propofol [5]. Ekmekci et al. reported that propofol-remifentanil is better than meperidine-midazolam concerning cognitive function in patients under sedation for colonoscopy [6]. Propofol-remifentanil allows earlier cognitive recovery than propofol-dexmedetomidine [7]. These results showed that propofol-remifentanil may be a good choice of anesthesia in elderly surgical patients.

However, both propofol and remifentanil have a rapid onset, short duration, and rapid recovery, which leads to early postoperative catheter-related bladder discomfort (CRBD) following urological procedures and earlier demand for postoperative analgesics [7, 8]. The clinical study indicated that the infusion of dexmedetomidine without a loading dose administered intraoperatively provided smooth and hemodynamically stable emergence and improved the quality of recovery with fewer postoperative side effects and analgesic requirements after nasal surgery [9]. Thus, we postulated that an intraoperative infusion of dexmedetomidine without a loading dose would decrease the incidence and severity of early postoperative CRBD and have little effect on cognitive dysfunction in elderly patients anesthetized with propofol-remifentanil. Therefore, we designed this study to test the hypothesis that remifentanil-propofol combined with or without dexmedetomidine would have the same effects on postoperative cognitive function in elderly patients.

## Methods

Following approval by the Ethics Committee of the Affiliated Hospital of North Sichuan Medical College, we obtained written informed consent from all the participants for this randomized prospective clinical trial conducted at the Affiliated Hospital of North Sichuan Medical College on patients with upper urinary tract calculi. This prospective, double-blind, randomized controlled study was registered at the Chinese Clinical Trial Registry (http://www.chictr.org.cn/; registration number: ChiCTR1900021254).

One hundred twenty adult ASA I–II patients between 60 and 75 years of age undergoing ureteroscopic holmium laser lithotripsy were enrolled in the study between February 2019 and September 2019. Patients scheduled for elective ureteroscopic holmium laser lithotripsy under general anesthesia and who fasted for 12 h without solid food and 6 h without clear liquids before the study were included. The exclusion criteria were as follows: history of adverse responses to propofol, remifentanil, or dexmedetomidine; the presence of cardiovascular disease, endocrine disease, and liver or kidney dysfunctions; smoking within 2 weeks; history of chronic use of alcohol, sedatives, and opioids; cognitive dysfunction; and change in surgical plan.

Patients were divided randomly into two groups, using sealed envelopes indicating the allocation, to receive intravenous dexmedetomidine 0.4 μg kg^−1^ h^−1^ (PRD group, *n* = 60) or intravenous normal saline, and the infusion rate of normal saline was set as 0.4 μg kg^−1^ h^−1^dexmedetomidine (in fact, dexmedetomidine was not administered) (PR group, *n* = 60) after general anesthesia induction. Randomization was performed by an anesthesiologist who was not responsible for the patients’ surgical anesthesia or data collection. The study drugs were administered by an anesthetic nurse, while the anesthesiologist responsible for the patient did not know what they were. The primary outcome was the score of the Mini-Mental State Examination. The secondary outcomes were the scores of the Richmond Agitation Sedation, Mini-Mental State Examination, State-Trait Anxiety Inventory, and Zung Self-Rating Depression Scale; memory recall of Arabic numerals; the duration of surgery; and the time to spontaneous respiration, recovery, and extubation.

Preoperative visits and communications with patients and their relatives were conducted the day before surgery. The patients were familiar with the questionnaires, which included the Mini-Mental State Examination (MMSE), State-Trait Anxiety Inventory (STAI), and Zung Self-Rating Depression Scale (ZSDS), and remembered five random Arabic numbers.

Patients enrolled in the study were premedicated with an intramuscular injection of atropine (0.5 mg) 30 min before the induction of anesthesia. When the patients arrived in the operating room (T_0_), the Arabic numbers (RAM) that were remembered the day before surgery were recalled, and the correct number was counted. Then, the Mini-Mental State Examination (MMSE), State-Trait Anxiety Inventory (STAI), and Zung Self-Rating Depression Scale (ZSDS) were applied. Routine monitoring included electrocardiography, noninvasive blood pressure (systolic blood pressure, mean arterial pressure, and diastolic blood pressure), heart rate, respiratory rate, pulse oximetry, end-tidal CO_2_, bispectral index, and temperature.

Patients were induced with intravenous propofol 2 mg/kg, remifentanil 2 μg/kg, and cisatracurium 0.15 mg/kg. After the endotracheal tube was inserted, controlled mechanical ventilation was adjusted to maintain an end-tidal carbon dioxide concentration of 40 to 45 mmHg. Immediately after the induction of anesthesia, dexmedetomidine 0.4 μg kg^−1^ h^−1^ was infused intravenously in the PRD group, while normal saline was administered in the PR group. Anesthesia was maintained with a plasma target concentration of propofol 2~3 μg/ml in the Marsh model and remifentanil 4~6 ng/ml in the Minto model by TCI (TCI-III-B Infusion Pump, Guangxi Willy Ark Technology Co., Ltd., China), and the value of the bispectral index was maintained between 40 and 60 during surgery. Cisatracurium was given intraoperatively if required. The administration of cisatracurium, dexmedetomidine or placebo, and propofol-remifentanil were stopped 45, 30, and 5 min before the end of the surgery, respectively. Bradycardia (heart rate below 50 beats/min) and hypotension (SBP below 90 mmHg) were treated with atropine (0.5 mg) and ephedrine (5 mg) intravenously, respectively.

Patients were extubated postoperatively after spontaneous respiration (tidal volume > 6 ml/kg, respiratory rate > 13/min), a train-of-four (TOF) ratio ≥ 0.9, SpO_2_ > 90% under air inspiration, and BIS > 80. The duration of surgery and time to spontaneous respiration, recovery, and extubation (time from stopping the administration of propofol-remifentanil to spontaneous respiration, recovery, and extubation, respectively) were recorded. Patients were transferred to the postanesthesia care unit (PACU) after extubation. O_2_ was applied at 5 l min^−1^ via a nasal catheter. The PACU emergence agitation score was evaluated 10 min (t_1_), 20 min (t_2_), 30 min (t_3_), and 60 min (t_4_) after extubation by an anesthetic nurse blinded to the study using the Richmond Agitation Sedation Score (RASS: +4, combative;+3, very agitated; +2, agitated; +1, restless; 0, alert and calm; -1,drowsy; -2, light sedation; -3, moderate sedation; -4, deepsedation; -5, unarousable) [10]. Emergence agitation (EA)was defined as any RASS score ≥+2, with severe EA defined as RASS ≥+3. When the modified Aldrete scorewas >9, the patients were transferred to the surgical ward [11]. The duration of stay in the PACU was recorded.

The MMSE, STAI, ZSDS, and RAM were applied at 3 h (T_1_), 6 h (T_2_), 24 h (T_3_), 48 h (T_4_), and 72 h (T_5_) postoperatively.

### Statistical analysis

A previous study [12] showed that the mean ± SD value of MMSE scores evaluated at 6 h postoperatively in patients anesthetized with intravenous propofol-remifentanil was 24.3 ± 2.3, and POCD was considered according to the criteria of MMSE score reductions of ≥ 1 ± standard deviation. We calculated that a sample size of 60 patients was required in each group at a power of 90%, with a two-sided significance level of 0.05 by an independent *t* test. To account for a 10% dropout rate, we included 69 patients in each group. We thus planned to enroll 138 subjects in this study.

Statistical analyses were performed using the SPSS 22.0 program. The results are expressed as the mean ± standard deviation (SD). One-way analysis of variance (ANOVA) was used to compare the mean differences between the groups for demographic data (age and weight), operation time, time to spontaneous respiration, time to recovery and time to extubation, and the dosage of propofol and remifentanil administered. Two-way ANOVA, followed by post hoc tests, was used to analyze the scores of MMSE, STAI, ZSDS, and RAM. The sex ratio, ASA physical status, levels of education, and RASS scores were analyzed using the *X*^2^ or Fisher’s exact tests. *P* < 0.05 was considered statistically significant.

## Results

**Fig. 1:**
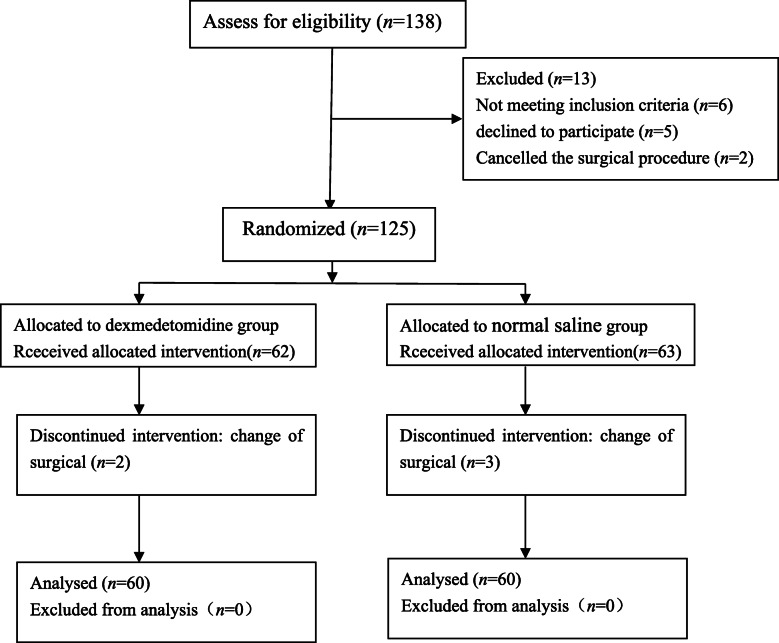
Study flow diagram

One hundred thirty-eight patients were screened for eligibility, six patients with hypertension were excluded, five patients declined to participate, and two patients were removed from the surgical procedure. A total of 125 patients were subsequently allocated to the two groups. The surgical plan was changed in five patients during the operation. A total of one hundred twenty patients completed the study and were analyzed (Fig. 1). There was no difference between the groups regarding demographics (Table 1). The duration of surgery, time to spontaneous respiration, and length of PACU stay time were similar between the groups. The dosage of propofol or remifentanil was significantly lower in the PRD group than in the PR group (*P* < 0.001). The time to recovery and tracheal extubation time were delayed more significantly in the PRD group (*P* < 0.001) (Table 2).

**Table 1:** Demographic data and patient characteristics were similar between the two groups

Patient characteristics	PR group, *n* = 60	PRD group, *n* = 60	*F*/*X*^2^ values	*P* values
Sex (male/female)	35/25	33/27	4	0.2615
Level of education (illiterate/primary school literacy)	27/33	29/31	2	0.1573
Age (years)	66.7 ± 4.1	65.6 ± 3.4	2.387	0.125
Weight (kg)	56.5 ± 5.4	55.8 ± 5.9	0.465	0.496
ASA (I/II)	20/40	18/42	2	0.1573

Values are mean ± SD, number of patients

*ASA* American Society of Anesthesiologists, *PR* propofol-remifentanil, *PRD* propofol-remifentanil and dexmedetomidine

**Table 2:** Clinical characteristics in the two groups. The duration of surgery, the time to spontaneous respiration, and the length of stay in the PACU were similar in the two groups. The dosage of propofol or remifentanil was significantly higher in the PRD group than in the PR group (*P <* 0.001). The time to recovery and tracheal extubation time were delayed greater in the PRD group compared with the PR group (*P <* 0.001)

Clinical characteristics	PR group, *n* = 60	PRD group, *n* = 60	*F* values	*P* values
Duration of surgery (min)	93.4 ± 10.9	94.1 ± 10.9	0.124	0.725
Dosage of propofol (mg)	517.0 ± 75.8	335.8 ± 40.8*	265.928	0.000
Dosage of remifentanil (mg)	486.0 ± 53.2	439.4 ± 32.7*	33.407	0.000
Time to spontaneous respiration (min)	16.4 ± 2.2	17.1 ± 2.8	2.375	0.126
Time to recovery (min)	20.0 ± 2.4	24.5 ± 3.6*	64.195	0.000
Tracheal extubation time (min)	21.2 ± 2.7	25.4 ± 3.6*	53.371	0.000
PACU stay time (min)	66.2 ± 5.2	67.9 ± 6.5	2.544	0.113

Values are mean ± SD

*PR* propofol-remifentanil, *PRD* propofol-remifentanil and dexmedetomidine, *PACU* postanesthesia care unit

*P < 0.001 vs. the PR group

The Mini-Mental State Examination scores are shown in Table 3. Compared to T_0_, the MMSE scores were lower at T_1_ and T_2_ in the two groups. However, the MMSE scores were similar between the two groups at T_0_, T_1_, T_2_, T_3_, T_4_, and T_5_. The Richmond Agitation Sedation Scores are shown in Table 4. The RASS scores in the PRD group were significantly lower than those in the PR group at t_1_, t_2_, and t_3_ (*P* < 0.001). The incidence of EA was significantly lower in the PRD group than in the PR group at t_1_ (10.0% [6/54] vs. 40.0% [24/36]), t_2_ (3.3% [2/58] vs. 40.0% [24/36]), and t_3_ (0.0% [0/60] vs. 23.3% [14/46]). The Zung Self-Rating Depression Scale scores are shown in Table 5. The ZSDS scores were higher in the PR group than in the PRD group at T_1_ and T_2_ (*P* < 0.001), and the ZSDS scores were similar between the two groups at T_0_, T_3_, T_4_, and T_5_. The ZSDS scores were higher at T_1_and T_2_ than at T_0_ in the PR group (*P* < 0.001), and there were no differences in ZSDS scores at T_0_, T_1_, T_2_, T_3_, T_4_, and T_5_ in the PRD groups (*P* = 0.359). The State-Trait Anxiety Inventory scores are shown in Table 6. The STAI scores were higher at T_1_ andT_2_ than at T_0_ in the PR group, and the STAI scores were higher at T_1_ than at T_0_ in the PRD group. The STAI scores at T_1_ and T_2_ were lower in the PRD group than in the PR group (*P* < 0.005). The recalled Arabic numbers are shown in Table 7. Compared to T_0_, the recalled Arabic numbers were lower at T_1_ and T_2_ in the PR group and at T_1_ in the PRD group (*P <* 0.001). The number of recalled Arabic numbers was lower at T_2_ in the PR group than in the PRD group (*P <* 0.001).

**Table 3:** The Mini-Mental State Examination scores at T_0_, T_1_, T_2_, T_3_, T_4_, and T_5_ in the two groups. The MMSE scores were lower at T_1_ and T_2_ compared to T_0_ in the two groups (*P <* 0.001). The MMSE scores were similar between the two groups at T_0_, T_1_, T_2_, T_3_, T_4_, and T_5_ (*P >* 0.05)

Time points	PR group	PRD group	*F* values	*P* values
T_0_	25.4 ± 2.2	25.5 ± 2.5	0.431	0.700
T_1_	20.7 ± 2.1*	21.3 ± 2.5*	0.423	0.183
T_2_	22.8 ± 2.0*	23.3 ± 2.0*	0.367	0.223
T_3_	25.0 ± 2.2	25.2 ± 2.6	0.432	0.644
T_4_	25.1 ± 2.2	25.2 ± 2.4	0.421	0.664
T_5_	25.3 ± 2.2	25.4 ± 2.5	0.429	0.756
*F* values	129.34	105.55	–	–
*P* values	0.000	0.000	–	–

Values are mean ± SD

*PR* propofol-remifentanil, *PRD* propofol-remifentanil and dexmedetomidine, *T*_*0*_ before the induction of anesthesia, *T*_*1*_ 3 h after surgery, *T*_*2*_ 6 h after surgery, *T*_*3*_ 24 h after surgery, *T*_*4*_ 48 h after surgery, *T*_*5*_ 72 h after surgery

*P < 0.001 vs. T_0_

**Table 4:** The Richmond Agitation Sedation scores at 10, 20, 30, and 60 min after extubation in the two groups. The RASS scores in the PRD group were significantly lower than that in the PR group at t_1_, t_2_, t_3_(*P <* 0.001). The incidence of EA was significantly lower in the PRD group than in the PR group at t1 (10.0% [6/54] vs. 40.0% [24/36]), t2 (3.3% [2/58] vs. 40.0% [24/36]), t3 (0.0% [0/60] vs. 23.3% [14/46])

Time points	Group	− 1	0	1	2	3	*X*^2^	*P* values
t_1_	PR	0	10	26	14	10	42.329	0.000*
	PRD	12	25	17	6	0		
t_2_	PR	0	17	19	16	8	54.262	0.000*
	PRD	21	23	14	2	0		
t_3_	PR	5	13	28	14	0	48.415	0.000*
	PRD	14	38	8	0	0		
t_4_	PR	7	36	17	0	0	2.930	0.231
	PRD	7	42	9	0	0		

Number of patients

*PR* propofol-remifentanil, *PRD* propofol-remifentanil and dexmedetomidine, *t*_*1*_ 10 min after extubation, *t*_*2*_ 20 min after extubation, *t*_*3*_ 30 min after extubation, *t*_*4*_ 60 min after extubation

*P < 0.001 vs. the PR group

**Table 5:** The Zung Self-Rating Depression Scale scores at T_0_, T_1_, T_2_, T_3_, T_4_, and T_5_ in the two groups. The ZSDS scores in the PRD group were lower compared to the PR group at T_1_ and T_2_ (*P <* 0.001), and the ZSDS scores were similar between the two groups at T_0_, T_3_, T_4_, and T_5_. The ZSDS scores were higher at T_1_ and T_2_ compared to T_0_ in the PR group (*P <* 0.001), and there were no differences in ZSDS scores at T_0_, T_1_, T_2_, T_3_, T_4_, and T_5_ in the PRD groups (*P =* 0.359)

Groups	Number	T_0_	T_1_	T_2_	T_3_	T_4_	T_5_	*F* values	*P* values
PR	60	43.2 ± 2.2	48.1 ± 2.5*	46.2 ± 2.4*	43.6 ± 1.6	43.2 ± 1.7	43.3 ± 1.9	69.06	0.000
PRD	60	42.8 ± 2.4	43.4 ± 2.2^#^	43.2 ± 1.7^#^	42.9 ± 2.0	42.7 ± 2.2	42.8 ± 1.70	1.35	0.248
*F* values		0.419	0.434	0.379	0.329	0.357	0.333	–	–
*P* values		0.362	0.000	0.000	0.028	0.138	0.136	–	–

Values are mean ± SD

*PR* propofol-remifentanil, *PRD* propofol-remifentanil and dexmedetomidine, *T*_*0*_ before the induction of anesthesia, *T*_*1*_ 3 h after surgery, *T*_*2*_ 6 h after surgery, *T*_*3*_ 24 h after surgery, *T*_*4*_ 48 h after surgery, *T*_*5*_ 72 h after surgery

*P < 0.001 vs. T_0_

^#^*P* < 0.005 vs. the PR group

**Table 6:** The State-Trait Anxiety Inventory scores at T_0_, T_1_, T_2_, T_3_, T_4_, and T_5_ in the two groups. The STAI scores were higher at T_1_ and T_2_ compared to T_0_ in the PR group, and the STAI scores were higher at T_1_ compared to T_0_ in the PRD group. The STAI scores at T_1_ and T_2_ were lower in the PRD group compared to the PR group (*P* < 0.005)

Indexes	Groups	Number	T_0_	T_1_	T_2_	T_3_	T_4_	T_5_	*F* values	*P* values
S-AI	PR	60	38.9 ± 3.4	48.6 ± 3.6*	45.3 ± 3.7*	39.0 ± 3.0	39.1 ± 3.1	39.1 ± 3.4	171.05	0.000
	PRD	60	39.5 ± 2.6	45.5 ± 2.5*^#^	39.9 ± 2.3^#^	39.9 ± 2.3	39.5 ± 2.3	39.6 ± 2.0	36.30	0.000
	*F* values	0.554	0.561	0.565	0.490	0.504	0.507	–	–	
	*P* values	0.294	0.000	0.000	0.079	0.509	0.266	–	–	
T-AI	PR	60	40.4 ± 2.9	46.9 ± 3.1*	45.9 ± 2.9*	40.7 ± 2.5	40.5 ± 2.5	40.7 ± 2.5	74.79	0.000
	PRD	60	41.1 ± 2.2	45.1 ± 3.1*^#^	41.4 ± 1.9^#^	40.8 ± 2.8	40.6 ± 2.2	40.7 ± 2.4	45.99	0.000
	*F* values	0.468	0.565	0.449	0.490	0.436	0.447	–	–	
	*P* values	0.179	0.002	0.000	0.812	0.789	1.000	–	–	

Values are mean ± SD

*PR* propofol-remifentanil, *PRD* propofol-remifentanil and dexmedetomidine, *S-AI* State Anxiety Inventory, *T-AI* State-Trait Anxiety Inventory, *T*_*0*_ before the induction of anesthesia, *T*_*1*_ 3 h after surgery, *T*_*2*_ 6 h after surgery, *T*_*3*_ 24 h after surgery, *T*_*4*_ 48 h after surgery, *T*_*5*_ 72 h after surgery

*P < 0.001 vs. T_0_

^#^*P* < 0.005 vs. the PR group

**Table 7:** The recalled Arabic numbers at T_0_, T_1_, T_2_, T_3_, T_4_, and T_5_ in the two groups. The recalled Arabic numbers were lower at T_1_ and T_2_ in the PR group and at T_1_ in the PRD group compared to T_0_ (*P <* 0.001). The number of recalled Arabic numbers was lower at T_2_ in the PR group than in the PRD group (*P <* 0.001)

Group	Number	T_0_	T_1_	T_2_	T_3_	T_4_	T_5_	*F* values	*P* values
PR	60	4.4 ± 0.6	2.7 ± 0.7*	3.1 ± 0.7*	4.2 ± 0.7	4.3 ± 0.6	4.3 ± 0.7	61.79	0.000
PRD	60	4.3 ± 0.7	2.6 ± 0.8*	4.1 ± 0.7^#^	4.3 ± 0.7	4.2 ± 0.6	4.3 ± 0.7	55.09	0.000
*F* values		0.118	0.134	0.126	0.127	0.114	0.122	–	–
*P* values		0.573	0.323	0.000	0.694	0.465	1.000	–	–

Values are mean ± SD

*PR* propofol-remifentanil, *PRD* propofol-remifentanil and dexmedetomidine, *S-AI* State Anxiety Inventory, *T-AI* State-Trait Anxiety Inventory, *T*_*0*_ before the induction of anesthesia, *T*_*1*_ 3 h after surgery, *T*_*2*_ 6 h after surgery, *T*_*3*_ 24 h after surgery, *T*_*4*_ 48 h after surgery, *T*_*5*_ 72 h after surgery

*P < 0.001 vs. T_0_

^#^*P* < 0.001 vs. the PR group

## Discussion

The results of this clinical trial showed that although there was no difference between the groups in influence on postoperative cognitive dysfunction, the patients anesthetized with remifentanil-propofol combined with or without dexmedetomidine had transient postoperative cognitive dysfunction. The combination of intravenous dexmedetomidine 0.4 μg kg^−1^ h^−1^ resulted in a lower incidence and severity of postoperative emergence agitation in elderly patients undergoing ureteroscopic holmium laser lithotripsy. The durations of anxiety and depression after the operation were longer in the patients treated without intravenous dexmedetomidine. Intravenous dexmedetomidine reduced the dose of remifentanil and propofol used during the operation.

Postoperative cognitive dysfunction (POCD) is one of the most common postoperative complications in elderly patients and is associated with an increased risk of death in the first year after surgery [13]. Advancing age, multiple surgeries, duration of anesthesia, and acute postoperative pain have been implicated as risk factors for POCD [14, 15]. Elderly surgical patients are likely to experience cognitive impairment preoperatively, and the impairment is associated with the development of delirium postoperatively [16]. We previously found that the incidence of postoperative cognitive dysfunction was higher in elderly patients who received inhalational anesthesia than in those with total intravenous anesthesia [17]. Both anesthetic and pain management strategies appear to influence the risk of cognitive dysfunction after an elective joint arthroplasty, and perioperative pain may be a risk factor for postoperative delirium [18–20]. In the present study, we selected patients undergoing ureteroscopic holmium laser lithotripsy with minor acute postoperative pain, thus avoiding the effects of postoperative pain and postoperative analgesic drugs on POCD in elderly patients. Our study showed that the MMSE scores were decreased significantly 3 h after surgery and were restored to the preanesthesia level 24 h after surgery in the two groups. This suggested that postoperative cognitive dysfunction was temporary during TIVA with remifentanil and propofol given by TCI with or without dexmedetomidine in elderly patients undergoing ureteroscopic holmium laser lithotripsy. In contrast, İlvan et al. [19] reported that the TIVA method did not affect postoperative early cognitive functions in either elderly or young patients who underwent lumbar disk surgery. The time to perform the MMSE postoperatively might be responsible for the differences. Because they performed the MMSE 24 h after surgery and found that no one experienced POCD, the MMSE scores were decreased significantly at the 3rd hour and restored at the 24th hour after surgery in our study. Therefore, their results could not demonstrate that no transient cognitive dysfunction occurred within 24 h postoperatively. Our results were consistent with previous studies showing that propofol might cause POCD [21, 22] and different from the results reported by Zhang et al. [23] showing that propofol might inhibit the inflammatory response in the central nervous system and improve POCD. This indicated that prolonged use of propofol-based general anesthesia may influence the central nervous system and that short-term infusion of propofol during TIVA would minimally influence postoperative cognitive function.

Dexmedetomidine is a new α_2_ adrenergic receptor agonist with a short elimination half-life (approximately 2 h), and it has dose-dependent sedative and analgesic effects and no negative effects on respiration [24]. A meta-analysis performed by Tan and Ho [25] found that dexmedetomidine could induce sedation without increasing delirium. These findings are compatible with the results of our study which found that dexmedetomidine has no effects on postoperative cognitive function in elderly patients anesthetized with remifentanil and propofol. Several studies have demonstrated that dexmedetomidine may benefit cognitive function in elderly patients due to its neuroprotective effect and anti-inflammatory properties [12, 24, 26]. In our study, we did not detect plasma inflammatory factors, and the effect of dexmedetomidine on plasma inflammatory mediators in elderly patients was unclear. Tan and Ho [25] reported that the risk of bradycardia was significantly higher when both a loading dose and high maintenance doses (> 0.7 μg kg^−1^ h^−1^) were used. In the present study, no patients required intervention for bradycardia or hypotension. This may be due to the intraoperative infusion of dexmedetomidine without a loading dose and lower maintenance doses (0.4 μg kg^−1^ h^−1^ < 0.7 μg kg^−1^ h^−1^).

Emergence agitation develops in the early phase of general anesthesia recovery and is observed more frequently in ENT (ear, nose, and throat) surgical patients [27]. EA is characterized by agitation, confusion, disorientation, and possible violent behavior [27, 28]. EA can cause hemorrhage, falling out of the bed, self-extubation, removal of catheters, and even injury to the patient or medical staff [29]. The present study describes a higher incidence of EA in the PR group than in the PRD group. This finding indicated that intravenous dexmedetomidine after induction of anesthesia significantly reduced the incidence and severity of postoperative EA in elderly patients undergoing TIVA with remifentanil and propofol for ureteroscopic holmium laser lithotripsy. Independent risk factors for EA, such as younger age, recent smoking, sevoflurane anesthesia, postoperative pain of NRS ≥ 5, presence of a tracheal tube, and presence of a urinary catheter, were identified [27]. In our study, postoperative catheter-related bladder discomfort (CRBD) was more common in elderly patients in the PR group. This suggested that intraoperative intravenous dexmedetomidine could decrease the incidence and severity of early postoperative CRBD. Our results were consistent with previous studies showing that intraoperative administration of dexmedetomidine is a safe and effective practice for the prevention of CRBD after lumbar microdiscectomy and can reduce postoperative pain [30–32]. The lower incidence of EA in the PRD group in the present study may be attributed to the decreased incidence and severity of early postoperative CRBD.

In our study, both the ZSDS scores and the STAI scores were increased at the 3rd hour and restored at the 12th hour after the operation in the PR group. However, only the STAI scores were increased at the 3rd hour and restored at the 6th hour postoperatively in the PRD group. This finding indicated that intraoperative infusion of dexmedetomidine could attenuate the severity of postoperative anxiety and depression in elderly patients. Ingrid Rundshagen reported that an anxious, depressed basal mood has been identified as an additional risk factor for POCD [33]. Then, the administration of dexmedetomidine may benefit postoperative cognitive function by decreasing the incidence and severity of postoperative anxiety and depression.

There are limitations to our study. Firstly, we did not detect plasma inflammatory factors in our study. Many studies have reported that the protective effect of dexmedetomidine on cognitive function is due to its anti-inflammatory properties [24–26]. Secondly, the patients selected in our study had almost no postoperative pain, so the effects of remifentanil-propofol combined with or without dexmedetomidine on cognitive dysfunction in elderly patients with postoperative pain were unclear. Thirdly, several clinical studies have found that anxiety and depression can lead to cognitive impairment in patients [34–36]. In our study, we found that intraoperative infusion of dexmedetomidine could attenuate the severity of postoperative anxiety and depression in elderly patients who had no preoperative anxiety or depression. Future studies should examine whether intraoperative infusion of dexmedetomidine may benefit postoperative cognitive function in patients with depressive symptomatology or anxiety symptomatology. Finally, the period time for POCD assessment (72 h) was relatively short, and the effect of intraoperative infusion of dexmedetomidine on postoperative long-term cognitive function in elderly patients was unclear.

In conclusion, the present study showed that intravenous dexmedetomidine could provide clinically relevant benefits in elderly patients undergoing ureteroscopic holmium laser lithotripsy. Dexmedetomidine administration could reduce the dosage of remifentanil and propofol needed during surgery. Although dexmedetomidine had no effect on postoperative cognitive dysfunction, it could reduce the incidence and severity of postoperative emergence agitation, anxiety, and depression in elderly patients.

### Assistance with the study

None.

### Presentation

None.

## Data Availability

We presented the study datasets in additional supporting files (PRD-PR study data. XLSX).
